# The Study of the Safety and Effectiveness of Motiva SmoothSilk Silicone Gel-Filled Breast Implants in Patients Undergoing Primary and Revisional Breast Augmentation: Three-Year Clinical Data

**DOI:** 10.1093/asj/sjae134

**Published:** 2024-10-01

**Authors:** Caroline Glicksman, Andrew Wolfe, Patricia McGuire

## Abstract

**Background:**

Silicone breast implant design has evolved over the last 50 years. Regulatory bodies including the FDA require data to support the modifications designed to improve the safety, efficacy, longevity, and biocompatibility of breast implants.

**Objectives:**

The authors reviewed the 3-year data on the safety and effectiveness of Motiva (Establishment Labs Holdings, Inc., Alajuela, Costa Rica) SmoothSilk silicone gel-filled breast implants submitted to the FDA. The current submitted data include the primary breast augmentation and revisional augmentation cohorts.

**Methods:**

The Motiva IDE is a prospective, single-arm, multicenter, 10-year pivotal study in which data are collected on breast augmentation, reconstruction, and revisional surgery. Three-year data were submitted to the FDA on adverse events, reoperations, patient and physician satisfaction, connective tissue diseases, and quality of life validated instruments. A subset of the patients underwent annual magnetic resonance imaging (MRI) at years 1, 2, and 3 to screen for implant rupture.

**Results:**

A total of 451 patients were implanted in the primary augmentation cohort and 109 patients in the revisional augmentation cohort. There were 218 patients enrolled in the MRI cohort. Reported rates for reoperation for any reason were 6.1% in the primary augmentation cohort (92.4% follow-up) and 25.8% in the revisional augmentation cohort (88.7% follow-up).

**Discussion:**

Motiva implants were first introduced in 2010. The 3-year Motiva data suggests that the leading cause of revisional surgery has shifted from capsular contracture and rupture to more subjective indications for reoperation such as malposition and size change.

**Conclusions:**

Three-year data from the primary augmentation and revisional augmentation cohorts submitted to the FDA demonstrate the safety and efficacy of the Motiva implants. There were low complication rates for implant-related complications and high surgeon and patient satisfaction.

**Level of Evidence: 2:**

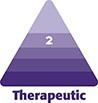

Establishment Labs Holdings, Inc. (Alajuela, Costa Rica) is a global medical technology company. The original prototypes for the Motiva current breast implant technology were developed 20 years ago in Costa Rica. Design and development activities were finalized with the launch of the Motiva Round implants in 2010. In 2011 the company obtained a CE mark, and in 2012 the second generation of Motiva Round implants was launched with changes in the gel and the addition of a new barrier technology (BluSeal) to reduce potential silicone leakage. In 2014, the Motiva Ergonomix implant was introduced. From 2019 to 2023, Establishment Labs was approved to market Motiva implants in more than 85 countries.

The US pivotal study included 2 styles of Motiva breast implants, the Motiva SmoothSilk implant with ProgressiveGel Plus (referred to as round) and the Motiva SmoothSilk Ergonomix ProgressiveGel Ultima implants (referred to as Ergonomix). All devices in the study were filled with the same sourced medical-grade long-term implantable silicone manufactured by Avantor-NuSil Silicone Technology (Carpinteria, CA) but mixed in different ratios to alter their rheological properties. All study implants were manufactured with the SmoothSilk surface and patch and contained a pigmented barrier layer (BluSeal). The pigmented barrier layer was purported to facilitate quality control during manufacture and to allow visual inspection of the implants by surgeons before implantation. The pigment represented less than 0.05% of the barrier layer material (NuSil Silicone Technologies). TrueMonobloc was the proprietary mechanism of bonding the shell, patch, and gel. Investigator surgeons could select implants with or without a microtransponder which would respond with a unique identification number when interrogated externally with a handheld device. This number coulc then be cross-referenced with Establishment Labs records to obtain device-related information as to implant type, surface, manufacturing date, serial number, and, if the device was registered, the date of implantation. This Motiva Q Inside Safety Technology (RFID) consisted of a passive microtransponder that was embedded in the silicone near the patch of the implant. It measured approximately 2 mm × 9 mm and was previously cleared by the FDA with the de Novo process (K033440; 21 CRF §880.6300) as an implantable device.^1^

Establishment Labs has developed a proprietary method of manufacturing the surface of the Motiva SmoothSilk shell. The shell surface is produced with a 3-dimensional (3D) inverted negative imprinting technology directly on the polydimethylsiloxane material.^2^ This is described as mandrel imprinting, in which the mandrel's surface architecture is transferred to the silicone during curing and the shell is subsequently turned inside out. In the last few years attention has been focused on shell properties that may affect the complex tissue interactions at the surface, including the ingrowth of soft tissues, the inflammatory response, and the ability of bacteria to adhere to the surface of the device.^3-5^ The SmoothSilk surface of the implant has a uniform surface with an average roughness of 4 microns that can be considered a modification of a smooth surface (average roughness of approximately 1 micron). The International Organization for Standardization (ISO) is currently updating their surface classification to include surface complexity. This classification scheme is based on the texturing technique, surface roughness, surface complexity (measure of added surface area compared to completely smooth surface), and pore size of the breast implant outer surface, as measured by scanning electron microscopy (SEM; Appendix, located online at www.aestheticsurgeryjournal.com).

## METHODS

The investigational device exemption (IDE) for the Motiva study on the safety and effectiveness of the Motiva silicone SmoothSilk gel-filled breast implants in patients undergoing primary breast augmentation, primary breast reconstruction, and revisional surgery was submitted to the FDA July 2017 and approved in March 2018. The study design was posted on clinicaltrials.gov (NCT03579901) in July 2018 and last updated on March 27, 2023. All patients signed an informed consent, and the study was designed following the principles of the Declaration of Helsinki. The study was IRB approved in 2018 through the Center for IRB Intelligence (CIRBI), Advarra, Columbia, MD. All approved investigators and their sites were required to notify the IRB of any unanticipated problems involving risks to patients or others, adverse device effects, and protocol deviations that might affect patient rights or the completeness and accuracy of the results. Twenty-five investigator sites and investigators underwent site training before patient enrollment, and the first patient was enrolled in the study on April 17, 2018. The study included patients aged 22 years and over for primary breast augmentation to increase breast size and revisional procedures to correct or improve the results of a previous breast augmentation procedure. Study indications for breast reconstruction included the replacement of breast tissue removed due to cancer, trauma, or severe breast abnormality and revisional surgery to correct or improve the original reconstructive procedure.

Study enrollment was completed in the augmentation cohorts on July 26, 2019, with a total of 451 patients implanted in the primary augmentation cohort and 109 patients in the revisional augmentation cohort. As part of the Motiva trial, a magnetic resonance imaging (MRI) substudy was designed to include up to 250 patients who would undergo a breast MRI scan at 1, 2, 3, 5, 7, and 10 years post surgery. MRI designated sites were selected in close proximity to the investigator's office to further encourage follow-up. Participation in the MRI subgroup was offered to all eligible patients, with compensation for their time at fair market value (FMV). A subgroup of 218 patients from the primary and revisional augmentation cohorts was enrolled. Patients could be discontinued from the MRI cohort due to claustrophobia concerns or a patient's decision to discontinue while remaining enrolled in the larger study.

Clinical data were collected at each investigator site on standardized paper case report forms at baseline, 3 to 6 weeks, and scheduled annual yearly follow-up visits. Data underwent double data entry in validated electronic data capture. Optional virtual patient monitoring became available in April 2020, a necessary modification of the protocol that allowed patients and investigators to complete annual patient follow-up during the peak of the COVID-19 pandemic.^6^

Patient demographic data were collected, along with previous lactation history, menopausal status, and history of connective tissue diseases (CTD). CTD signs and symptoms were recorded at each scheduled time point to evaluate if prevalence increased with exposure to breast implants. Self-reported measures of health were recorded with the SF-36v2 Health Survey (2000). The BREAST-Q (version 2.0) was added to the protocol in July 2021 and will continue to collect data through 10 years.

A screening baseline mammogram was required for all patients over the age of 35 before study enrollment. Any postoperative mammography results were evaluated, and Kaplan-Meier analysis estimated the cumulative incidence of new postoperative abnormal mammograms (defined as BI-RADS 4 suspicious abnormality). In addition to standardized photography at baseline and all follow-up visits, 3D imaging (Vectra XT 3D Imaging System, Canfield Scientific, Parsippany, NJ; Divina 3-D Imaging System, Establishment Labs, Alajuela, Costa Rica) was an additional study requirement. The Vectra 3D scans continue to be required on all patients in the primary augmentation cohort, while the optional Divina was discontinued in 2020.

**Figure 1.**
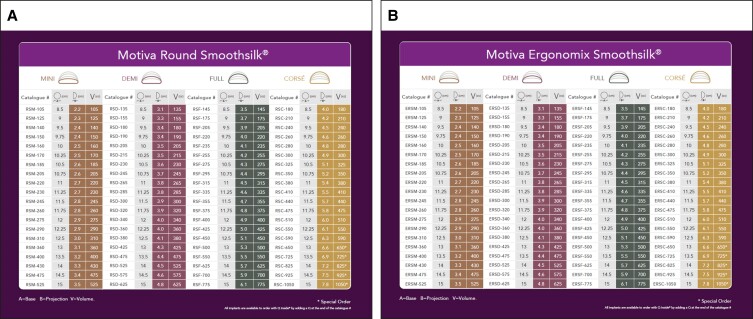
(A) Motiva SmoothSilk Round and (B) Motiva Ergonomix implants.

The study investigators were able to select from the 2 styles of Motiva implants available, the SmoothSilk Ergonomix and the SmoothSilk Round, in consultation with their patients, and determined the optimal style, size, and fill for each patient (Figure 1). Surgical procedural data collected on the day of surgery included incision approach, pocket location, the use of funnels, placement of drains, the device style and volume, and specifics of pocket irrigation.

To maintain high patient compliance throughout the 10-year study, patients receive compensation at FMV for their annual scheduled follow-ups. Financial incentives have been shown to create higher patient retention in both device and pharmaceutical clinical trials.^7^ Patients who were explanted without reimplantation were asked to remain in the study and continue their annual follow-up visits out to 10 years. The MRI subpopulation included patients in both augmentation cohorts, with MRI scheduled at years 1, 2, 3, 5, 7, and 10 years for the analysis of rupture. In addition, a subgroup of patients were further defined by the presence or absence of the microtransponder for safety and effectiveness.

### Data Collection and Statistical Analysis

#### Safety Analysis

The safety population included all patients implanted with a Motiva device. Analysis was conducted by cohort and performed at both the patient and implant levels. Safety assessment included Kaplan-Meier analysis of any patient complications, adverse events, and reoperations with 95% confidence intervals. Additional gathered information included analysis of rupture, CTD signs and symptoms, breast imaging including mammography and ultrasound when indicated, breast cancer, and breast implant–associated anaplastic large cell lymphoma (BIA-ALCL). CTD signs and symptoms were not collected at baseline before implantation, but patients with previously diagnosed CTD were excluded from the study. A Cox regression analysis was performed for complications, reoperations, and adverse events.

Explanatory variables included age, race, smoking status, surgical approach, surgical placement, incision size, irrigation solutions (including antibiotic irrigation and Betadine [Purdue Pharma, Stamford, CT]), pocket location, implant volume, and type of implant selected.

#### Effectiveness Analyses

Effectiveness was based on a performance goal, with the endpoint measured on a 5-point Likert scale rating the degree of patient satisfaction or dissatisfaction. Quality of life assessments and surgeon satisfaction with outcome were calculated at each time point. The BREAST-Q was added to the effectiveness analysis in 2021 and therefore does not capture all patients at baseline enrolled in the augmentation cohorts.

### Monitoring During COVID Pandemic

In 2020, investigational sites were faced with unprecedented challenges due to the COVID pandemic and subsequent restrictions placed upon the healthcare system that varied from state to state. A virtual clinical trial visit plan was submitted to the FDA in March 2020 and promptly accepted; this permitted video conferencing for patients and study sites in lockdown. Sites were instructed to adhere to virtual platforms that were HIPAA compliant. Data points that were unable to be collected during virtual follow-up visits and/or procedures that were not performed due to the virtual nature of the visit (digital photography, 3D digital imaging, MRI, and in some instances measurements) were reported in the source documentation. These deviations were not considered to increase the risks or affect the well-being of patients and data points.

## RESULTS

### Demographics, Follow-up by Cohort, and Procedural Data

Final enrollment included 451 primary augmentation and 109 revisional augmentation female patients, with a total enrollment of 560 patients. Patient compliance with annual follow-up through 3 years was 92.4% in the primary augmentation cohort and 88.7% in the revisional augmentation cohort (Supplemental Tables 1, 2, located online at www.aestheticsurgeryjournal.com).

The age range for primary augmentation patients was 22 to 69 years (mean, 33.5) and for revisional augmentation 22 to 69 years (mean 44.5). Most patients were White, had a college education or higher degree, and were married. (Supplemental Table 3, located online at www.aestheticsurgeryjournal.com).

Both SmoothSilk Ergonomix and SmoothSilk Round implants were included in the device trial, and investigators selected SmoothSilk Ergonomix implants more often in both the primary augmentation cohort (88.7%) and revisional augmentation cohort (87.2%). Surgeons were also given the choice of whether or not to select implants with a radiofrequency identification device (RFID). In the primary augmentation cohort, 30% of implants included the RFID, and in the revisional augmentation cohort 17.4% of implants included the RFID. Implant volumes ranged from 105 to 1050 cc, with a mean volume of 331 cc in the primary augmentation cohort and 409 cc in the revisional augmentation cohort.

The most common incision site for implant insertion in the primary augmentation cohort was the inframammary fold (IMF) (85.3%), followed by transaxillary (9.5%). In the revisional augmentation cohort, an inframammary approach was performed most often (81.7%), followed by periareolar (10.1%), mastopexy (6.4%), and transaxillary (1.8%). In the augmentation cohort, the most frequent pocket location was partial submuscular (dual-plane) (94.6%), and subglandular placement was rare (2.2%). The revisional augmentation cohort surgeons also favored the partial submuscular (dual-plane) (78.9%), but subglandular placement was less uncommon (17.4%).

Comprehensive data collected on the day of surgery included details of pocket irrigation. Some combination of pocket irrigation was employed by 93.8% of investigator surgeons in the primary augmentation cohort and 100% of the surgeons in the revisional augmentation cohort. The most common irrigation solution was the combination of Betadine and triple antibiotics in both the primary augmentation cohort (55.4%) and the revisional augmentation cohort (63.3%). Betadine mixed with saline was the next most frequent irrigation solution in both the primary augmentation cohort (17.1%) and the revisional augmentation cohort (18.3%). Other combinations of antibiotics and saline were utilized in the primary augmentation cohort (5.5%) and revisional augmentation cohort (4.6%). Normal saline alone was an irrigant in the primary augmentation cohort (0.7%) and in the revisional augmentation cohort (2.8%). Drains were rarely placed in either the primary (2%) or revisional (14%) augmentation cohorts. Funnels for implant insertion were also documented for primary augmentation (72%) and for revisional augmentation (61%) (Tables 1, 2).

**Table 1.** Operative Characteristics (Procedural Data) of Primary and Revisional Augmentation Cohorts by Implant

Surgical characteristics	Primary augmentation (*n* = 901)	Revisional augmentation (*n* = 218)	Overall augmentation (*n* = 1119)
Device Style			
Round	102 (11.3%)	28 (12.8%)	130 (11.6%)
Ergonomix	799 (88.7%)	190 (87.2%)	989 (88.4%)
Radiofrequency identification device (RFID)			
With RFID	270 (30.0%)	38 (17.4%)	308 (27%)
Without RFID	631 (70%)	180 (82.6%)	811 (72.5%)
Implant volume			
105-300 cc	378 (42%)	58 (26.6%)	436 (39.0%)
305-500 cc	502 (55.7%)	114 (52.3%)	616 (55.0%)
505-800 cc	21 (2.3%)	40 (18.3%)	61 (5.5%)
805-1050 cc	0	6 (2.8%)	6 (0.5%)
Incision site			
Inframammary	769 (85.3%)	178 (81.7%)	947 (84.6%)
Mastopexy	20 (2.2%)	14 (6.4%)	34 (3.0%0
Periareolar	26 (2.9%)	22 (10.1%)	48 (4.3%)
Transaxillary	86 (9.5%)	4 (1.8%)	90 (8.0%)
Placement			
Complete muscle coverage	24 (2.7%)	4 (1.8%)	28 (2.5%)
Partial submuscular-dual plane	852 (94.6%)	172 (78.9%)	1024 (91.5%)
Subfascial	5 (0.6%)	4.0 (1.8%)	9 (0.8%)
Subglandular	20 (2.2%)	38 (17.4%)	58 (5.2%)
Incision length (cm)			
Mean (SD)	4.2 (1.69)	5.7 (3.14)	4.5 (2.12)
Median (min-max)	4.0 (2-17)	5.0 (3-30)	4.0 (2-30)
Pocket irrigation^a^			
No pocket irrigation	28 (6.2%)	0	28 (5.0%)
At least 1 irrigation	423 (93.8%)	109 (100%)	532 (95%)
Antibiotics only	8 (1.8%)	0	8 (1.4%)
Antibiotics/saline	25 (5.5%)	5 (4.6%)	30 (5.4%)
Antibiotics/Betadine/saline	250 (55.4%)	69 (63.3%)	319 (57.0%)
Betadine/saline	77 (17.1%)	20 (18.3%)	97 (17.3%)
Other antiseptic (not Betadine)/saline	44 (9.8%)	1 (0.9%)	45 (8.0%)
Other antiseptic only (not Betadine)	3 (0.7%)	0	3 (.5%)
Saline only	3 (0.7%)	3 (2.8%)	6 (1.1%)

^a^Sum of individual irrigation types may be greater than total because a single patient may have multiple irrigants. Max, maximum; min, minimum; SD, standard deviation.

**Table 2.** Operative Characteristics (Procedural Data) of Primary and Revisional Augmentation Cohorts by Patient

Surgical characteristics	Primary augmentation (*n* = 451)	Revisional augmentation (*n* = 109)	Overall augmentation (*n* = 560)
Funnel use during surgery			
Funnel	325 (72.1%)	66 (60.6%)	391 (69.8%)
No funnel	126 (27.9%)	43 (39.4%)	169 (30.2%)
Drain placement during surgery			
Both breasts	7 (1.6%)	15 (13.8%)	22 (3.9%)
Right breast	0	3 (2.8%)	3 (0.5%)
Left breast	0	1 (0.9%)	1 (0.2%)
No drains	444 (98.4%)	90 (82.6%)	534 (95.4%)

There were 218 patients enrolled overall in the MRI substudy, 176 patients (352 implants) in the primary augmentation cohort and 42 patients (84 implants) in the revisional augmentation cohort. At year 3, 25 patients exited the study due to claustrophobia or other patient decisions, with 88.2% follow-up. At year 3 there were 1 confirmed rupture in the primary augmentation cohort and 0 confirmed ruptures in the revisional augmentation cohort.

### Complications and Reoperations

Kaplan-Meier risk analysis at 3 years for local breast complications was reported by patient and by implant. In the primary augmentation cohort, reported breast complications at 3 years included size change (6.9%), implant malposition (3.2%), infection (0.9%), hematoma (0.7%), breast pain (0.7%), suspected or confirmed implant rupture (0.6%), capsular contracture grade III or IV (0.5%), glandular ptosis (0.2%), animation deformity (0.2%), and asymmetry (0.2%). In the revisional augmentation cohort, reported complications at 3 years included size change (20.7%), capsular contracture grade III or IV (6.7%), implant malposition (4.9%), glandular ptosis (4.8%), asymmetry (3.9%), hematoma (1.8%), and infection (0.9%). Explantation without replacement occurred in 1 patient (0.2%) in the primary augmentation cohort and 3 patients (2.9%) in the revisional augmentation cohort. There was 1 confirmed rupture in the primary augmentation cohort, and there were no late seromas (Table 3).

**Table 3.** Kaplan-Meyer Risk Analysis at Year 3 by Patient

Event	Primary augmentation (*n* = 451)	Revisional augmentation (*n* = 109)
Reoperation	27 (6.1%)	27 (25.8%)
Explantation	7 (1.6%)	17 (16.5%)
Explantation with replacement	6 (1.4%)	14 (13.8%)
Explantation without replacement	1 (0.2%)	3 (2.9%)
Number of patients with at least 1 reoperation	27 (6.0%)	27 (24.8%)
Risk of any complication^a^	37 (8.4%)	30 (28.4%)
Breast pain	3 (0.7%)	1 (1.0%)
Animation deformity	1 (0.2%)	0
Asymmetry	1 (0.2%)	4 (3.9%)
Breast cancer (new)	0	0
Capsular contracture grade II with surgical intervention	1 (0.2%)	2 (1.9%)
Capsular contracture III/IV	2 (0.5%)	7 (6.7%)
BIA-ALCL	0	0
Double capsule	0	1 (1.0%)
Infection	4 (0.9%)	1 (0.9%)
Rupture suspected/confirmed	1 (0.6%)	0
Size change	2 (6.9%)	6 (20.7%)
Delayed wound healing	1 (0.2%)	0
Hematoma	3 (0.7%)	2 (1.8%)
Iatrogenic injury to implant	0	1 (1.0%)
Implant extrusion	0	1 (1.0%)
Malposition	14 (3.2)	5 (4.9%)
Palpability/visibility	1 (0.2%)	0
Breast mass/cyst/lump	1 (0.2%)	2 (2.4%)
Ptosis	1 (0.2%)	5 (4.8%)
Nipple complication	1 (0.2%)	0
Skin rash	1 (0.2%)	0
Seroma (late >1 year)	0	0
Wrinkling/rippling	2 (0.5%)	0

^a^Does not include complications assessed as mild severity, except for capsular contracture III/IV, implant extrusion, and suspected or confirmed rupture. BIA-ALCL, breast implant–associated anaplastic large cell lymphoma.

Patients with a history of CTD or rheumatologic illness were excluded from enrollment. Enrolled patients were asked to document any new onset of CTD or rheumatologic illness or symptoms at each study time point. There were no new reported cases of either CTD or rheumatologic illness in either the primary or revisional augmentation cohorts (Table 4).

**Table 4.** Risk of Nonlocal Complications: Connective Tissue Diseases and Rheumatologic Illness

Complication/Time point	Number of events (cumulative)	Kaplan-Meier risk	95% CI
Primary breast augmentation			
Rheumatic disease, any^a^			
Week 6	0 (0)	—	
Year 1	0 (0)	—	
Year 2	0 (0)	—	
Year 3	0 (0)	—	
Rheumatoid arthritis			
Week 6	0 (0)	—	
Year 1	0 (0)	—	
Year 2	0 (0)	—	
Year 3	0 (0)	—	
Systemic lupus			
Week 6	0 (0)	—	
Year 1	0 (0)		
Year 2	0 (0)	—	
Year 3	0 (0)	—	
Other rheumatologic			
Week 6	0 (0)	—	
Year 1	0 (0)	—	
Year 2	0 (0)	—	
Year 3	0 (0)	—	
Revisional breast augmentation			
Rheumatic disease, any^a^			
Week 6	0 (0)	—	
Year 1	0 (0)	—	
Year 2	0 (0)	—	
Year 3	0 (0)	—	
Rheumatoid arthritis			
Week 6	0 (0)	—	
Year 1	0 (0)	—	
Year 2	0 (0)	—	
Year 3	0 (0)	—	
Systemic lupus			
Week 6	0 (0)	—	
Year 1	0 (0)	—	
Year 2	0 (0)	—	
Year 3	0 (0)	—	
Other rheumatologic			
Week 6	0 (0)	—	
Year 1	0 (0)	—	
Year 2	0 (0)	—	
Year 3	0 (0)	—	

^a^Rheumatologic and connective tissue diseases include rheumatoid arthritis, systemic lupus erythematosus, discoid lupus, scleroderma, vasculitis, dermatomyositis, Raynaud's phenomenon, Sjogren's syndrome, CREST syndrome, carpal tunnel syndrome, multiple sclerosis–like syndrome, multiple myeloma–like syndrome, chronic fatigue syndrome, fibromyalgia. CI, confidence interval.

At 3 years there were 6 patients discontinued from the primary augmentation cohort: 1 unrelated death, 1 patient explanted and replaced with a nonstudy device, 2 requested to be discontinued, and 2 were lost to follow-up. In the revisional augmentation cohort, there were 5 patients discontinued: 1 unrelated death, 2 explanted with nonstudy device replacement, 2 requested to be discontinued, and 0 were lost to follow-up.

In the primary augmentation cohort, there were 29 reoperations in 27 patients (6.0%) at 3 years. The indications for explantation with or without replacement for these 29 reoperations included reoperation for malposition in 13 patients (44.8%), grade III/IV capsular contracture in 3 patients (10.3%), size change in 2 patients (6.9%), and infection in 2 patients (6.9%). In the revisional augmentation cohort, there were 29 reoperations in 27 patients (24.8%) over the 3-year period. The indications for these 29 reoperations included revision for size change in 6 patients (20.7%), grade III/IV capsular contracture in 6 patients (20.7%), glandular ptosis in 5 patients (17.2%), and hematoma in 4 patients (13.8%). Animation deformity as a cause for reoperation was rare, occurring in 1 patient in the primary augmentation cohort (Table 5).

**Table 5.** Primary Reason for Reoperation

Primary reason for reoperation^a^	Primary augmentation (*n* = 29)	Revisional augmentation (*n* = 29)	Overall augmentation (*n* = 58)
Infection	2 (6.9%)	1 (3.4%)	3 (5.2%)
Capsular contracture	3 (10.3%)	6 (20.7%)	9 (15.5%)
Implant extrusion	0	1 (3.4%)	1 (1.7%)
Hematoma	3 (10.3%)	4 (13.8%)	7 (12.1%)
Breast pain	0	1 (3.4%)	1 (1.7%)
Implant malposition	13 (44.8%)	2 (6.9%)	15 (25.9%)
Upper pole fullness	0	1 (3.4%)	1 (1.7%)
Animation deformity	1 (3.4%)	0	1 (1.7%)
Asymmetry	1 (3.4%)	1 (3.4%)	2 (3.4%)
Ptosis	1 (3.4%)	5 (17.2%)	6 (10.3%)
Hypertrophic/abnormal scarring	2 (6.9%)	1 (3.4%)	3 (5.2%)
Mass/cyst/lump	1 (3.4%)	0	1 (1.7%)
Size change/patient choice	2 (6.9%)	6 (20.7%)	8 (13.8%)

^a^If multiple reasons were provided, then primary was assigned in the order that it appears in the table. Primary reasons that were not reported (eg, rupture) do not appear on this table.

### BIA-ALCL, BIA-SCC, Breast Cancer, and Patient Deaths

There were no new reported breast cancers in either augmentation cohort at 3 years. There were also no reports of either BIA-ALCL or breast implant–associated squamous cell carcinoma (BIA-SCC) in either of the breast augmentation cohorts at year 3. There was 1 reported death in the primary breast augmentation cohort as a complication of rectal cancer at year 2 and 1 death at year 1 in the revisional augmentation cohort due to trauma.

### Clinical Effectiveness and Patient Satisfaction

Clinical effectiveness in this study was measured based upon patient and physician satisfaction gathered at each scheduled study visit, based on a 5-point Likert scale (Table 6). Patient satisfaction at 3 years for primary augmentation was (97.1%) and for revisional augmentation was (87.5%), yielding high overall patient satisfaction at year 3 (95.4%). Physician satisfaction was very high at year 3 for both primary augmentation (99.0%) and revisional augmentation (95.5%). Patient-reported satisfaction measures were also documented with the BREAST-Q for both primary and revisional augmentation cohorts (Table 7).

**Table 6.** Patient Satisfaction: 5-Point Likert Scale at Year 3

Satisfaction assessment^a^	Primary augmentation (*n* = 410)	Revisional augmentation (*n* = 88)	Overall augmentation
Patient satisfaction			
Satisfaction response			
Very satisfied	332 (81.0%)	64 (72.7%)	396 (79.5%)
Satisfied	66 (16.1%)	13 (14.8%)	79 (15.9%)
Neither dissatisfied nor satisfied	5 (1.2%)	5 (5.7%)	10 (2.0%)
Dissatisfied	5 (1.2%)	4 (4.5%)	9 (1.8%)
Very dissatisfied	2 (0.5%)	2 (2.3%)	4 (0.8%)
Satisfaction summary			
Satisfied	398 (97.1%)	77 (87.5%)	475 (95.4%)
Neither	5 (1.2%)	5 (5.7%)	10 (2.0%)
Dissatisfied	7 (1.7%)	6 (6.8%)	13 (2.6%)
Satisfaction analysis			
Percentage satisfied	97.1%	87.5%	95.4%
95% confidence interval	(94.9%, 98.5%)	(78.7%, 93.6%)	(93.2%, 97.1%)
Investigator satisfaction			
Satisfaction response			
Very satisfied	364 (88.8%)	72 (81.8%)	436 (87.6%)
Satisfied	42 (10.2%)	12 (13.6%)	54 (10.8%)
Neither dissatisfied nor satisfied	3 (0.7%)	2 (2.3%)	5 (1.0%)
Dissatisfied	1 (0.2%)	1 (1.1%)	2 (0.4%)
Very dissatisfied	0	1 (1.1%)	1 (0.2%)
Satisfaction summary			
Satisfied	406 (99.0%)	84 (95.5%)	490 (98.4%)
Neither	3 (0.7%)	2 (2.3%)	5 (1.0%)
Dissatisfied	1 (0.2%)	2 (2.3%)	3 (0.6%)
Satisfaction analysis			
Percentage satisfied	99.0%	95.5%	98.4%
95% confidence interval	(97.5%, 99.7%)	(88.8%, 98.7%)	(96.9%, 99.3%)

^a^Data include only patients without missing responses for primary implants at this follow-up year 3.

**Table 7.** Satisfaction With Breast Implants Grom BREAST-Q at Year 3: Primary Augmentation and Revision Augmentation Subjects

	*n*	Dissatisfied	Satisfied
**Primary Augmentation**			
Amount of cleavage when wearing a bra	383	5.5%	94.5%
Bra fit	383	4.2%	95.8%
Breast firmness	383	4.7%	95.3%
Breast shape without a bra	383	6.8%	93.2%
Breast size	382	6.5%	93.5%
Breast size matches the rest of the body	382	3.4%	96.6%
Breast feels to touch	382	2.4%	97.6%
How close breasts are when not wearing a bra	383	11.7%	88.3%
How close breasts match with each other	383	3.9%	96.1%`
Implant evenness/position relative to each other	381	5.0%	95.0%
Implant position on the chest	383	7.8%	92.2%
Look in the mirror clothed	383	3.7%	96.3%
Look in the mirror unclothed	381	10.5%	89.5%
Natural look of breasts	378	2.9%	97.1%
Naturally breasts sit/hang	380	6.8%	93.2%
**Revision Augmentation**			
Amount of cleavage when wearing a bra	76	7.9%	92.1%
Bra fit	76	7.9%	92.1%
Breast firmness	76	1.3%	98.7%
Breast shape without a bra	76	10.5%	89.5%
Breast size	76	7.9%	92.1%
Breast size matches the rest of the body	75	8.0%	92.0%
Breast feels to touch	76	1.3%	98.7%
How close breasts are when not wearing a bra	76	7.9%	92.1%
How close breasts match with each other	76	15.8%	84.2%
Implant evenness/position relative to each other	76	14.5%	85.5%
Implant position on the chest	76	6.6%	93.4%
Look in the mirror clothed	76	3.9%	96.1%
Look in the mirror unclothed	76	11.8%	88.2%
Natural look of breasts	76	10.5%	89.5%
Naturally breasts sit/hang	76	6.6%	93.4%

## DISCUSSION

Motiva breast implants were first introduced to the market in 2011 and have undergone several modifications since then. They are currently approved in over 85 countries. This report contains the 3-year data from the first prospective, multicenter clinical trial to study the safety and efficacy of the Motiva SmoothSilk Ergonomix silicone gel-filled breast implants and the Motiva SmoothSilk Round gel implants. The implants included in this core study are designed with several new brand-named technologies, including ProgressiveGel Ultima, TrueMonobloc, and BluSeal.

Investigator surgeons received both in-person and online training before patient enrollment. Investigator surgeons were selected based on their years in practice, support staff required to follow the study protocol, and their previous performance as clinical investigators in premarket approval (PMA) studies. Training included educational materials on the novel implant matrix and a series of videos and in-person meetings with European and Costa Rican surgeons with more than 5 years of clinical experience with Motiva implants.^2,8^ Investigator training was designed to reduce the potential learning curve associated with these investigational implants.^9^

At 3 years, the reoperation rate for any reason was 6.0% in the primary augmentation cohort and 24.8% in the revisional augmentation cohort. The reoperation rate was higher in the revisional augmentation cohort, consistent with previous core studies showing that revisional patients are at higher risk of additional surgical procedures.^10,11^ Six patients (6.7%) in the revisional augmentation cohort underwent reoperation for capsular contracture grade III/IV by year 3. Five of these 6 patients had either previous grade III/IV capsular contracture or a ruptured device. It should also be noted that the study protocol prohibited either acellular dermal matrix or Scaffold in any revisional augmentation procedure.

Surgeon investigators were required to document the irrigation agents utilized during surgery and specifically provide intraoperative details regarding antibiotic solutions, Betadine, and funnels at the time of implantation. Most investigators reported some contamination mitigation techniques in both primary and revisional augmentation. These mitigation strategies included an IMF approach in the primary augmentation cohort (85.3%) and revisional augmentation cohort (81.7%), and pocket irrigation by the surgeon reported as combinations of saline, Betadine, and antibiotics, overall 95.0%. Additionally, the investigator surgeons reported the use of funnels in the primary augmentation cohort (72.1%) and revisional augmentation cohort (60.6%), an absent or uncommon practice when previous manufacturer IDE studies were undertaken. These techniques have been shown to reduce pocket contamination and potentially reduce capsular contracture and have become increasingly incorporated into breast implant procedures.^12^

Previously published core studies on the safety and efficacy of silicone breast implants initiated 10 to 20 years ago reported high revision rates for the leading drivers of reoperation: capsular contracture, size change, and malposition.^13,14^ It is evident that changes in surgeon practices over the last 2 decades, including better preoperative patient education, more precise surgical planning, and modifications in surgical techniques that include steps to reduce pocket contamination, have led to improvements in patient outcomes, as demonstrated in more modern clinical trials.^15-17^ The 3-year Motiva data demonstrate that the leading reasons for revisional surgery in both primary and revisional breast augmentation surgery have shifted from an objective classification of capsular contracture (grade III or IV) to more subjective indications such as malposition and size change, for which both the physician and the patient may determine the necessity of a revisional procedure.

Implant malposition was an initial concern of the study investigators due to published descriptions of explanted Motiva capsules being thin and translucent.^4^ By year 3, the reported incidence of malposition was 3.2% in the primary augmentation cohort and 4.9% in the revisional augmentation cohort. However, malposition did account for 44.8% of the revisional surgeries in the primary augmentation cohort (*n* = 13). There were other parameters that required a short learning curve for the investigator surgeons, such as interpreting a new implant sizing matrix and understanding the performance of the 2 gel fills. The fundamentals such as patient selection, implant selection, pocket location, precise surgical dissection, and meticulous closure remained the essential steps in minimizing complications. Malposition was not associated with a learning curve based on a review of the timing of patient enrollment and the experience of the investigators.^2,18^

Implant malposition results from a combination of factors, including soft tissue quality, implant volume, pocket dissection, adjustments to the IMF, and the closure of the IMF. Additionally, supportive postoperative bras and limitations in postoperative exercise have been associated with a reduction in implant malposition.^19^ Revision for malposition is based in part on the management of patient expectations, because the range of malposition can vary from a very mild unilateral malposition to significant bilateral malposition. A potential limitation of the study protocol was the lack of defined quantitative guidelines that classified the degree of malposition or the type of malposition. Investigators were not required to report the gradation of malposition in centimeters, nor did they define whether the malposition was inferior, lateral, medial, or superior. Revision for malposition in primary augmentation occurred at only 6 of 25 investigator sites, and this included revision for several patients with superior malposition. Previous published studies from Europe, Asia, and South America have reported very thin capsule formation surrounding these implants.^20,21^ In a retrospective study by Randquist, inferior and lateral displacement occurred most often in the first 3 years after the transition to Motiva implants. It is important to note that the transition in their study was from macrotextured shaped implants to smooth round devices and required additional refinements in implant selection, pocket dissection, management of the IMF incision, and postoperative care.^2,7^ For most surgeons in the United States, the transition will be from smooth devices to Motiva.

## CONCLUSIONS

The 3-year data on Motiva implants demonstrate very low device-related complications. With high patient follow-up at 3 years (91.7%), the overall findings indicate that Motiva implants are safe and effective. In addition, patient and physician satisfaction was high and the risk of adverse events was low.

## Supplemental Material

This article contains supplemental material located online at www.aestheticsurgeryjournal.com.

## Supplementary Material

sjae134_Supplementary_Data
