# Supplementary material for: The Study of the Safety and Effectiveness of Motiva SmoothSilk Silicone Gel-Filled Breast Implants in Patients Undergoing Primary and Revisional Breast Augmentation: Three-Year Clinical Data
Source: Aesthet Surg J. 2024 Oct 1;44(12):1273–85. doi: 10.1093/asj/sjae134 (PMC11565863; doi:10.1093/asj/sjae134)
Supplement: sjae134_Supplementary_Data [file sjae134_supplementary_data.zip › Appendix 1-Implant Surface Finish Definitions-ISO.docx]

**Appendix 1:ISO Classification on Implant Surface Finish Technique^1^**

Three categories of surface finish techniques to the outer surface of the implant:

1. No Intentional texturization (NTX)
2. Additional shell surface coating layer, including but not limited to:

- Polyurethane foam layer (PUL)
- Other material layer (OML)

1. Intentionally textured silicone shell surfaces, including but not limited to:

- Salt loss-closed: salt, usually sodium chloride, applied to uncured silicone and an extra layer of uncured silicone is applied over the salt, which is abraded after curing to remove the salt, (SLC);
- Salt loss-open: salt, usually sodium chloride, is applied to uncured silicone and the salt is washed away after curing;
- Other crystal loss: closed similar to salt loss but with other crystal, (CRC);
- Other crystal loss: open: same as open salt loss but with other crystal (CRO);
- Gas diffusion- sub-surface: ammonium carbonate is embedded in the silicone and the gases bubble through the uncured silicone during curing (GDD);
- Gas diffusion-surface: ammonium carbonate is on the surface of the silicone and leaves grain shaped openings on the silicone surface when thermally decomposes during curing (GDS);
- Polyurethane imprinting: polyurethane is pressed onto the uncured silicone and removed before curing,(PUI);
- Mandrel imprinting: the mandrel is textured, and the texture is transferred to the silicone during curing (shell then turned inside out), (MAI); and
- Other techniques, (OTH).

-------------------------------------------------------------------------------------------------

1. ISO/DIS 14607:2023(E); ISO/TC 150

Secretariat: DIN, Non-active surgical implants-Mammary Implants-Scientific Requirements

Fourth edition, Date: 2023-11-01
