# Supplementary material for: The Study of the Safety and Effectiveness of Motiva SmoothSilk Silicone Gel-Filled Breast Implants in Patients Undergoing Primary and Revisional Breast Augmentation: Three-Year Clinical Data
Source: Aesthet Surg J. 2024 Oct 1;44(12):1273–85. doi: 10.1093/asj/sjae134 (PMC11565863; doi:10.1093/asj/sjae134)
Supplement: sjae134_Supplementary_Data [file sjae134_supplementary_data.zip › Supplemental Table_1-Subject_Follow-up_by_Cohort.docx]

**Supplemental Table 1.** Participant Follow-up Compliance: Primary and Revision Augmentation and MRI Cohorts

| Participant follow-up, primary and revision augmentation cohorts | By subject | | |
| --- | --- | --- | --- |
| Accounting by window | Primary augmentation (n=451) | Revision augmentation (n=109) | Overall augmentation (n=560) |
| Week 3-6 |  |  |  |
| Due | 451 | 109 | 560 |
| Discontinued |  |  |  |
| Deaths | 0 | 0 | 0 |
| Explanted with non-study replacement | 0 | 0 | 0 |
| Subject request | 0 | 0 | 0 |
| Lost to follow-up | 0 | 0 | 0 |
| Other | 0 | 0 | 0 |
| Seen | 446 | 109 | 555 |
| Expected* | 451 | 109 | 560 |
| % Compliant out of expected | 98.9% | 100.0% | 99.1% |
| Year 1 |  |  |  |
| Due | 451 | 109 | 560 |
| Discontinued |  |  |  |
| Deaths | 0 | 1 | 1 |
| Explanted with non-study replacement | 1 | 0 | 1 |
| Subject request | 1 | 1 | 2 |
| Lost-to-follow-up | 0 | 0 | 0 |
| Other | 0 | 0 | 0 |
| Seen | 437 | 101 | 538 |
| Expected* | 450 | 108 | 558 |
| % Compliant out of expected | 97.1% | 93.5% | 96.4% |
| Year 2 |  |  |  |
| Due [1] | 451 | 109 | 560 |
| Discontinued |  |  |  |
| Deaths | 1 | 1 | 2 |
| Explanted with non-study replacement | 1 | 2 | 3 |
| Subject request | 2 | 2 | 4 |
| Lost-to-follow-up | 1 | 0 | 1 |
| Other | 0 | 0 | 0 |
| Seen | 428 | 94 | 522 |
| Expected* | 449 | 106 | 555 |
| % Compliant out of expected | 95.3% | 88.7% | 94.1% |
| Year 3 |  |  |  |
| Due | 451 | 109 | 560 |
| Discontinued |  |  |  |
| Deaths | 1 | 1 | 2 |
| Explanted with non-study replacement | 1 | 2 | 3 |
| Subject request | 2 | 2 | 4 |
| Lost-to-follow-up | 2 | 0 | 2 |
| Other | 0 | 0 | 0 |
| Seen | 415 | 94 | 509 |
| Expected | 449 | 106 | 555 |
| % Compliant out of expected | 92.4% | 88.7% | 91.7% |

* "Expected" indicates the number “Due” minus all discontinuations except subject decision.
