# Supplementary material for: The Study of the Safety and Effectiveness of Motiva SmoothSilk Silicone Gel-Filled Breast Implants in Patients Undergoing Primary and Revisional Breast Augmentation: Three-Year Clinical Data
Source: Aesthet Surg J. 2024 Oct 1;44(12):1273–85. doi: 10.1093/asj/sjae134 (PMC11565863; doi:10.1093/asj/sjae134)
Supplement: sjae134_Supplementary_Data [file sjae134_supplementary_data.zip › Supplemental Table_2-MRI_Follow-up_Compliance_Primary_and_Revision_Augmentation_Subjects.docx]

**Supplemental Table 2.** MRI Follow-up Compliance: Primary and Revision Augmentation Subjects

| MRI follow-up compliance- primary and revision augmentation cohorts | By subject | | |
| --- | --- | --- | --- |
| Accounting by window | Primary augmentation (n=176) | Revision augmentation (n=42) | Overall augmentation (n=218) |
| Year 1 |  |  |  |
| Due | 176 | 42 | 218 |
| Discontinued |  |  |  |
| Claustrophobic | 2 | 1 | 3 |
| Deaths | 0 | 0 | 0 |
| Primary devices removed | 1 | 0 | 1 |
| Unavailable* | 2 | 2 | 4 |
| Subject decision | 2 | 0 | 2 |
| Seen | 160 | 37 | 197 |
| Expected** | 171 | 39 | 210 |
| % Compliant out of expected | 93.6% | 94.9% | 93.8% |
| Year 2 |  |  |  |
| Due | 176 | 42 | 218 |
| Discontinued |  |  |  |
| Claustrophobic | 3 | 2 | 5 |
| Deaths | 0 | 0 | 0 |
| Primary devices removed | 2 | 4 | 6 |
| Unavailable* | 7 | 0 | 7 |
| Subject decision | 2 | 0 | 2 |
| Seen | 149 | 32 | 181 |
| Expected** | 164 | 36 | 200 |
| % Compliant out of expected | 90.9% | 88.9% | 90.5% |
| Year 3 |  |  |  |
| Due | 176 | 42 | 218 |
| Discontinued |  |  |  |
| Claustrophobic | 5 | 2 | 7 |
| Deaths | 0 | 0 | 0 |
| Primary devices removed | 2 | 5 | 7 |
| Unavailable* | 7 | 2 | 9 |
| Subject decision | 2 | 0 | 2 |
| Seen | 142 | 30 | 172 |
| Expected** | 162 | 33 | 195 |
| % Compliant out of expected | 87.7% | 90.9% | 88.2% |

* “Unavailable” includes subjects who were pregnant or who had other temporary medical conditions/circumstances preventing an MRI. These subjects will return to the denominator for compliance calculations after resolution of the medical condition/circumstance.

** "Expected" indicates the number “Due” minus all discontinuations except subject decision.
