# Supplementary material for: The Study of the Safety and Effectiveness of Motiva SmoothSilk Silicone Gel-Filled Breast Implants in Patients Undergoing Primary and Revisional Breast Augmentation: Three-Year Clinical Data
Source: Aesthet Surg J. 2024 Oct 1;44(12):1273–85. doi: 10.1093/asj/sjae134 (PMC11565863; doi:10.1093/asj/sjae134)
Supplement: sjae134_Supplementary_Data [file sjae134_supplementary_data.zip › Supplemental Table_3-Demographics_Motiva_IDE-docx.docx]

**Supplemental Table 3.** Demographics: Primary and Revision Augmentation by Subject

| Characteristics | Primary augmentation (N=451) | Revision augmentation (N=109) | Overall augmentation (N=560) |
| --- | --- | --- | --- |
| Race |  |  |  |
| American Indian or Alaska Native | 1 (0.2%) | 0 | 1 (0.2%) |
| Asian | 28 (6.2%) | 4 (3.7%) | 32 (5.7%) |
| Black | 9 (2.0%) | 0 | 9 (1.6%) |
| Native Hawaiian or Pacific Islander | 1 (0.2%) | 2 (1.8%) | 3 (0.5%) |
| White | 389 (86.3%) | 96 (88.1%) | 485 (86.6%) |
| Other | 23 (5.1%) | 7 (6.4%) | 30 (5.4%) |
| Ethnicity |  |  |  |
| Hispanic/ Latino | 37 (8.2%) | 12 (11.0%) | 49 (8.8%) |
| Not Hispanic/ Latino | 413 (91.6%) | 97 (89.0%) | 510 (91.1%) |
| Not reported | 1 (0.2%) | 0 | 1 (0.2%) |
| Education |  |  |  |
| Less than high school | 2.0 (0.4%) | 2 (1.8%) | 4 (0.7%) |
| High school/GED | 38 (8.4%) | 14 (12.8%) | 52 (9.3%) |
| Some college/Vocational school | 113 (25.1%) | 22 (20.2%) | 135 (24.1%) |
| College graduate | 202 (44.8%) | 52 (47.7%) | 254 (45.4%) |
| Post-graduate education | 96 (21.3%) | 19 (17.4%) | 115 (20.5%) |
| Occupation |  |  |  |
| Clerical/sales | 57 (12.6%) | 19 (17.4%) | 76 (13.6%) |
| Homemaker | 39 (8.6%) | 7 (6.4%) | 46 (8.2%) |
| Professional | 243 (53.9%) | 67 (61.5%) | 310 (55.4%) |
| Service | 41 (9.1%) | 5 (4.6%) | 46 (8.2%) |
| Student | 34 (7.5%) | 3 (2.8%) | 37 (6.6%) |
| Trade | 24 (5.3%) | 1 (0.9%) | 25 (4.5%) |
| Other | 13 (2.9%) | 7 (6.4%) | 20 (3.6%) |
| Marital Status |  |  |  |
| Divorced | 49 (10.9%) | 18 (16.5%) | 67 (12.0%) |
| Married | 218 (48.3%) | 60 (55.0%) | 278 (49.6%) |
| Separated | 5 (1.1%) | 1 (0.9%) | 6 (1.1%) |
| Single | 176 (39.0%) | 27 (24.8%) | 203 (36.3%) |
| Widowed | 3 (0.7%) | 3 (2.8%) | 6 (1.1%) |
